# P-345. Prevalence of Integrase HIV-1 Drug Resistance Mutations in the United States: 2019-2024

**DOI:** 10.1093/ofid/ofaf695.563

**Published:** 2026-01-11

**Authors:** Cassidy Henegar, Johnny Lai, Kimberley Brown, Bryn Jones, Annemiek de Ruiter, Gayathri Sridhar, Mark Underwood, Charles M Walworth, Vani Vannappagari

**Affiliations:** ViiV Healthcare, Chapel Hill, North Carolina; Monogram Biosciences, San Francisco, California; ViiV Healthcare, Chapel Hill, North Carolina; ViiV Healthcare, Chapel Hill, North Carolina; ViiV Healthcare., London, England, United Kingdom; ViiV Healthcare, Chapel Hill, North Carolina; ViiV Healthcare, Chapel Hill, North Carolina; Monogram Biosciences/LabCorp, Laguna Beach, CA; ViiV Healthcare, Chapel Hill, North Carolina

## Abstract

**Background:**

The integrase strand transfer inhibitor (INSTI) class of antiretrovirals (ARV), specifically 2nd generation INSTIs (dolutegravir, bictegravir, cabotegravir), has been increasingly used in HIV treatment due to high levels of effectiveness and tolerability, improved adherence, and high barriers to resistance. At the population level, patterns of ARV use can impact prevalence of HIV drug resistance mutations. This analysis used data from a large testing database to assess changes in INSTI resistance mutation frequencies and class-level susceptibility with expanding use in the United States 2019-2024.

**Figure:**
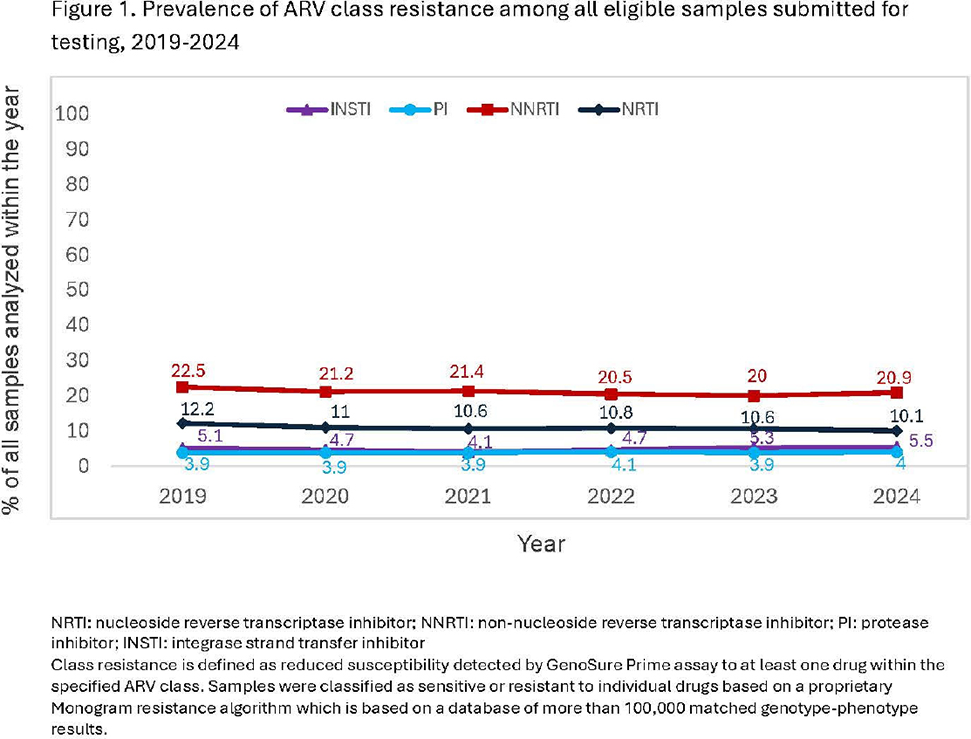

**Figure:**
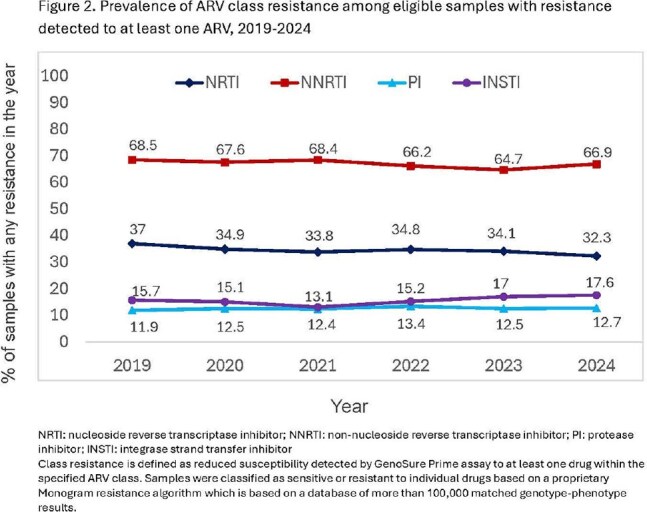

**Methods:**

Samples from adults with HIV-1 in the US and US territories submitted for routine genotypic resistance testing to the 4 major ARV classes (protease inhibitors, nucleoside reverse transcriptase inhibitor, non-nucleoside reverse transcriptase inhibitors and INSTIs) between January 1, 2019, and December 31, 2024, were analyzed. Class-level resistance was defined as reduced susceptibility to at least one drug within an ARV class; drug susceptibility was assessed using a paired genotype-phenotype algorithm that identified fold change thresholds for reduced sensitivity among individual mutations and drug exposures.

**Figure:**
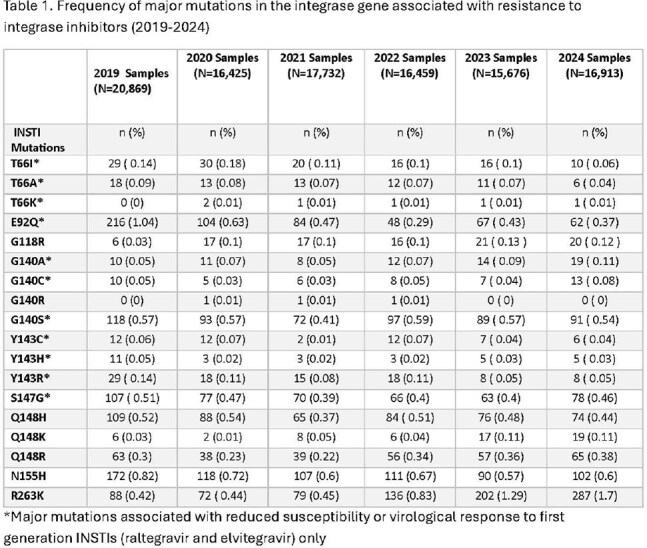

**Results:**

104,074 samples were evaluated. Frequency of INSTI resistance was low and stable (range: 4.1-5.5% of tested samples; Figure 1) across years. Among 32,761 (31.5%) samples with any resistance, 15.6% (n=5,099) demonstrated reduced susceptibility to ≥1 INSTI (range: 13.1-17.6%; Figure 2). Prevalence estimates for individual major mutations impacting the INSTI class were very low across all years (Table 1). Most were stable, with small declines in N155H (2019-2024: 0.82-0.6%) and some variants impacting first generation INSTIs [raltegravir, elvitegravir; E92Q (1.04-0.37%), T661 (0.14-0.06%, Y143R: 0.14-0.05%). R263K showed increasing frequency (0.42-1.7%) but remained uncommon.

**Conclusion:**

From 2019-2024, the prevalence of INSTI resistance in the US and US territories remained low despite increased and widespread use. Individual major INSTI mutations were infrequent. Observed trends reflect greater use of 2^nd^ generation INSTIs, which have low failure rates and high barriers to resistance.

**Disclosures:**

Cassidy Henegar, PhD, MSPH, ViiV Healthcare: Employee|ViiV Healthcare: Stocks/Bonds (Public Company) Johnny Lai, BS, Labcorp: Employee|Labcorp: Stocks/Bonds (Public Company) Kimberley Brown, PharmD, ViiV Healthcare: Employee|ViiV Healthcare: Stocks/Bonds (Public Company) Bryn Jones, MBChB, GSK: Stocks/Bonds (Public Company)|ViiV Healthcare: Employee Annemiek de Ruiter, MBBS FRCP, ViiV Healthcare: Stocks/Bonds (Public Company) Gayathri Sridhar, MBBS, MPH, PhD, GlaxoSmithKline: Stocks/Bonds (Public Company)|ViiV Healthcare: Full Time Employee Mark Underwood, PhD, ViiV Healthcare: Stocks/Bonds (Public Company) Charles M. Walworth, MD, Labcorp: Employee|Labcorp: Stocks/Bonds (Public Company)|Labcorp: Stocks/Bonds (Public Company) Vani Vannappagari, MBBS, MPH, PhD, ViiV Healthcare: Full time Employee of ViiV Healthcare and owns GSK stock|ViiV Healthcare: Stocks/Bonds (Public Company)

